# Transcriptome analysis of G protein-coupled receptors in distinct genetic subgroups of acute myeloid leukemia: identification of potential disease-specific targets

**DOI:** 10.1038/bcj.2016.36

**Published:** 2016-06-03

**Authors:** A Maiga, S Lemieux, C Pabst, V-P Lavallée, M Bouvier, G Sauvageau, J Hébert

**Affiliations:** 1 Institute for Research in Immunology and Cancer (IRIC), Université de Montréal, Montréal, Québec, Canada; 2 Department of Computer Science and Operations Research, Université de Montréal, Montréal, Québec, Canada; 3 Division of Hematology-Oncology, Maisonneuve-Rosemont Hospital, Montréal, Québec, Canada; 4 Department of Biochemistry, Faculty of Medicine, Université de Montréal, Montréal, Québec, Canada; 5 Leukemia Cell Bank of Quebec, Maisonneuve-Rosemont Hospital, Montréal, Québec, Canada; 6 Department of Medicine, Faculty of Medicine, Université de Montréal, Montréal, Québec, Canada

## Abstract

Acute myeloid leukemia (AML) is associated with poor clinical outcome and the development of more effective therapies is urgently needed. G protein-coupled receptors (GPCRs) represent attractive therapeutic targets, accounting for approximately 30% of all targets of marketed drugs. Using next-generation sequencing, we studied the expression of 772 GPCRs in 148 genetically diverse AML specimens, normal blood and bone marrow cell populations as well as cord blood-derived CD34-positive cells. Among these receptors, 30 are overexpressed and 19 are downregulated in AML samples compared with normal CD34-positive cells. Upregulated GPCRs are enriched in chemokine (*CCR1*, *CXCR4*, *CCR2*, *CX3CR1*, *CCR7* and *CCRL2*), adhesion (*CD97*, *EMR1*, *EMR2* and *GPR114*) and purine (including *P2RY2* and *P2RY13*) receptor subfamilies. The downregulated receptors include adhesion GPCRs, such as *LPHN1*, *GPR125*, *GPR56*, *CELSR3* and *GPR126*, protease-activated receptors (*F2R* and *F2RL1*) and the Frizzled family receptors *SMO* and *FZD6*. Interestingly, specific deregulation was observed in genetically distinct subgroups of AML, thereby identifying different potential therapeutic targets in these frequent AML subgroups.

## Introduction

G protein-coupled receptors (GPCRs) represent the largest family of membrane receptors with an estimated number of 800 members in human. They are key transducers that bind a vast diversity of ligands allowing the cells to adapt to their environment by regulating a wide variety of physiological processes including the control of blood pressure, heart rate, digestive processes, hormone secretion, cell growth and migration as well as vision and olfaction. Binding to their ligands leads to conformational rearrangements promoting the engagement and modulation of many distinct downstream signaling effectors that are both G protein-dependent and independent.^1, 2^

Several GPCRs are critical for cell proliferation and survival, and can be aberrantly expressed in cancer cells.^3, 4^ For example, PAR1 is overexpressed in invasive breast carcinomas^5^ or advanced-stage prostate cancer.^6, 7^ Likewise, the chemokine receptor CXCR4 has an important role in tumor metastasis and angiogenesis.^3, 4^ Moreover, the Wnt target gene, *Lgr5*, identified as a marker of intestinal stem cells, is implicated in mouse intestinal tumorigenesis^8, 9^ and its expression is also associated with poor clinical outcome in colorectal cancer.^10^

Although GPCRs are targets for approximately 30% of all marketed drugs,^11^ only a very limited number of agonists or antagonists acting through these receptors are currently used for cancer therapy.^12^ Notable exceptions include peptide antagonists of gonadotropin releasing-hormone receptor in prostate cancer,^12^ a somatostatin receptor agonist (Octreotide) and a growth hormone receptor antagonist (Pegvisomant) for neuroendocrine tumors,^12^ as well as Vismodegib, an antagonist of smoothened (SMO), approved by the US Food and Drug Administration (FDA) to treat advanced basal cell carcinoma.^13^

In acute myeloid leukemia (AML), CXCR4 overexpression has been associated with poor outcome.^14, 15, 16^ Moreover, *in vivo* studies have shown that the use of AMD3465, a small molecule antagonist of CXCR4, increases the mobilization of AML cells into the peripheral blood and improves the efficacy of chemotherapy.^17^ This activity has been explored in a phase 1/2 clinical study showing that the addition of CXCR4 antagonists to chemotherapy is possible in AML and might improve the remission rate.^18^ The role of GPCRs in mouse leukemic cells was also suggested in a transcriptome analysis of two related leukemia clones which differ in their stem cell frequency.^19^ This study revealed that genes encoding GPCRs were the most differentially expressed between the two clones compared with other classes of genes (~22% versus ~5%).

Despite these punctual observations, an exhaustive assessment of GPCR expression in human AML is lacking. To address this issue, we sequenced the transcriptome of a large cohort of AML samples and herein report the expression pattern of GPCRs in 148 AML samples.

## Materials and methods

### Human primary leukemic and cord blood cells

The 148 AML samples of the Leucegene cohort used for this study (described in Supplementary Table 1) were collected by the Banque de cellules leucémiques du Québec (BCLQ) with an informed consent and approval of the project by the Research Ethics Board of the Maisonneuve-Rosemont Hospital and Université de Montréal. The genetic subgroups of the AML samples included in this study are listed in Supplementary Table 2. Cord blood samples (*n*=12) collected with an informed consent were provided by Héma-Québec and pre-enriched for CD34^+^ cells before being sorted for the CD34 APC^+^/CD45RA PE^−^ cell population as previously described.^20^ The Cancer Genome Atlas (TCGA) RNA-Seq AML dataset has been downloaded from the TCGA website (https://tcga-data.nci.nih.gov/tcga/tcgaDownload.jsp) in November 2013 and the associated clinical informations obtained from Cancer Genome Atlas Research Network.^21^

### Sorting of normal bone marrow and peripheral blood cell populations

Three unsorted fresh bone marrow samples from healthy donors were purchased from Lonza (Lonza 1M-125; Basel, Switzerland). Red blood cell lysis was performed prior to resuspending the cells in phosphate-buffered saline, 0.1% bovine serum albumin and DNase 10 μg/ml. Subpopulations were sorted on a BD Aria II sorter (BD Biosciences, San Jose, CA, USA) based on published surface marker combinations (Supplementary Methods and Supplementary Table 3). Peripheral blood was collected from healthy donors with informed consent, subjected to red blood cell lysis and subsequently sorted on the basis of the following sorting strategy: Granulocytes (SSChigh, CD33^+^), B cells (Lymphocyte gate FSClow, SSClow, CD19^+^), T cells (Lymphocyte gate FSClow, SSClow, CD3^+^), Monocytes (Monocyte gate FSChigh, SSClow/med, CD14bright, CD33med), total white blood cells (WBC). Sorting purity was checked on aliquots after sorting, and cells were counted, resuspended in TRIzol reagent and stored at −80 °C until RNA isolation was performed according to the manufacturer's instructions. An additional purification step on RNeasy mini columns (Qiagen 74104; Hilden, Germany) was performed to optimize RNA quality, which was subsequently tested on an Agilent bioanalyzer 2100 (Agilent, Santa Clara, CA, USA). A minimum of 50 000 cells was used for RNA Sequencing (RNA-Seq), which was performed as described below.

### Sequencing and RNA-Seq data analysis

RNA-Seq was performed using an Illumina HiSeq 2000 instrument (Illumina, San Diego, CA, USA). Libraries were prepared according to the manufacturer's recommendations (Illumina). RefSeq annotations were based on the UCSC January 27th 2011 version. The alignment to reference genome (hg19) was carried out using the CASAVA 1.8.2 package and Eland v2 mapping software and bioinformatic analyses were performed as described earlier.^22^

### Statistical analysis

RNA-Seq data in Reads Per Kilobase per Million mapped reads (RPKM) were transformed to lRPKM (log_2_(RPKM+1)), where +1 was added to avoid excessive variations due to very small values. Log transformation was performed to avoid overrepresentation of extreme values. Highly expressed GPCRs were selected using a threshold of 3.5 lRPKM (or 10.35 RPKM) as shown in Supplementary Figure 1A. The variability of expression between samples was determined by calculating the coefficient of variation, a ratio between the standard deviation and the mean expression value. As illustrated in Supplementary Figure 1B, genes with a coefficient of variation smaller than the threshold (50%, gray area) are considered as GPCRs with low variability in their expression. Upregulated and downregulated GPCRs were described as those having a difference in median expression between AML and normal CD34^+^ cells greater than 1 and less than −1, respectively.

### GPCR subfamily enrichment analysis

Grouping of GPCR subfamilies was based on the International Union of Basic and Clinical Pharmacology (IUPHAR) database classification downloaded from the website in July 2014 (http://www.guidetopharmacology.org/). To complete the classification and subdivide the class A group into further subgroups, the GRAFS phylogenetic classification of GPCRs was also used.^23^ Taste receptors have been added along with vomeronasal receptors, opsins and three orphan GPCRs (*GPR137B*, *TAPT1* and *XPR1*). Overall, GPCRs were classified into 18 subfamilies (Supplementary Table 4).

The GPCR subfamily enrichment analysis in the upregulated or downregulated groups was performed using a Fisher's exact test. Significance (two-tailed *P*-value) was calculated using the function FET of the add-in Fisherexact downloaded from http://www.obertfamily.com/software/fisherexact.html. The receptors associated with a specific AML genetic subgroup were identified by calculating a difference in mean expression level (lRPKM) between samples with and without the genetic abnormality. An arbitrary difference of 1.5 lRPKM and a significant Student's *t* test (*P*-value<0.05) were used as cutoff levels to identify differentially expressed GPCRs in the different genetically defined subgroups of the studied AML cohort.

## Results

### GPCRs expression in human AML and normal CD34-positive cells

Using RNA-Seq, we have evaluated the expression of GPCRs in 148 AML samples and compared it with that observed in normal cord blood-derived CD34^+^CD45RA^−^ hematopoietic stem and progenitor cells (hereafter called CD34^+^ cells) and in normal bone marrow and peripheral blood cell populations. The 772 GPCRs analyzed in this study comprise all GPCRs included in the IUPHAR database, as well as 370 olfactory, 24 taste and 4 vomeronasal receptors. Information about the receptor subfamilies and the 18 subgroups based on their ligands is provided in Supplementary Tables 4, 5 and 6. Overall, 240 GPCRs were expressed at ⩾1 lRPKM (used as an arbitrary threshold) in at least one AML sample (Supplementary Table 5). Expression was above 3.5 lRPKM (highly expressed) for 111 and above 6.7 lRPKM (very highly expressed) for 19 receptors.

**Figure 1:**
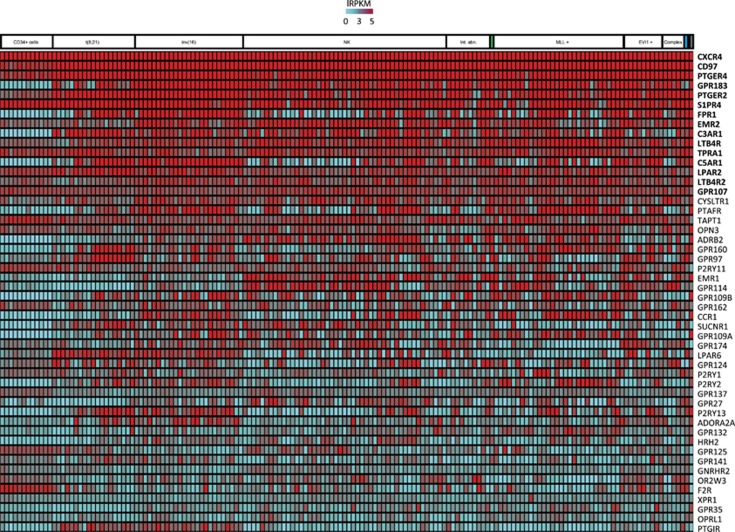
Identification of overexpressed GPCRs in AML. RNA-Seq was used to determine expression levels of 772 GPCRs in 148 AML samples of the Leucegene cohort and 12 samples of normal cord blood-derived CD34^+^ CD45RA^−^ cells. The 50 GPCRs with the highest median expression levels in AML samples are presented in the heatmap. The 15 GPCRs that have a higher expression level as defined in Supplementary Figure 1A are in bold. NK, normal karyotype; Int.abn., Intermediate abnormal karyotype; MLL+, AML with *MLL* translocations; EVI1+, AML with *EVI1* rearrangements; Complex, AML with three or more unrelated clonal chromosomal abnormalities. Boxes in green, blue or gray represent one sample with *NUP98-NSD1* fusion, 17p deletion or an insufficient number of metaphases respectively. RNA-Seq data were transformed to lRPKM (log_2_(RPKM+1)).

We first ranked the various GPCRs according to their median expression level from highest to lowest in AML (Figure 1) and in CD34^+^ cells (Supplementary Figure 2). Using the threshold of 3.5 lRPKM, the most highly expressed GPCRs in AML cells are in decreasing order: *CXCR4*, *CD97*, *PTGER4*, *GPR183*, *PTGER2*, *S1PR4*, *FPR1*, *EMR2*, *C3AR1*, *LTB4R*, *TPRA1*, *C5AR1*, *LPAR2*, *LTB4R2* and *GPR107*. This contrasts with the expression profiles observed in normal CD34^+^ cells where the rank order of expression is: *GPR56*, *CXCR4*, *S1PR4*, *HTR1F*, *F2R*, *TAPT1*, *PTGER4*, *CD97*, *GABBR1*, *TPRA1*, *LPAR2*, *SMO*, *P2RY11*, *LPHN1*, *GPR107* and *GPR126*. In addition to the different order of expression levels observed among highly expressed GPCRs, some receptors that are not found in the most highly expressed ones in normal CD34^+^ cells are clearly overexpressed in AML (Figure 1).

Real-time quantitative RT-PCR studies confirmed RNA-Seq results for 10 selected GPCRs and revealed a robust correlation between both methods (Supplementary Figure 3 and Supplementary Table 7). We also confirmed high protein expression levels of selected GPCRs for which validated antibodies were available, using flow cytometry as shown in Supplementary Figures 4 and 5. With the exception of CD97, which is uniformly highly expressed in all samples tested, GPCR expression was distributed unequally within each patient sample highlighting the possibility of defining AML subpopulations with these protein markers (Supplementary Figure 5).

### Deregulated GPCRs in AML belong to specific receptor subfamilies

**Figure 2:**
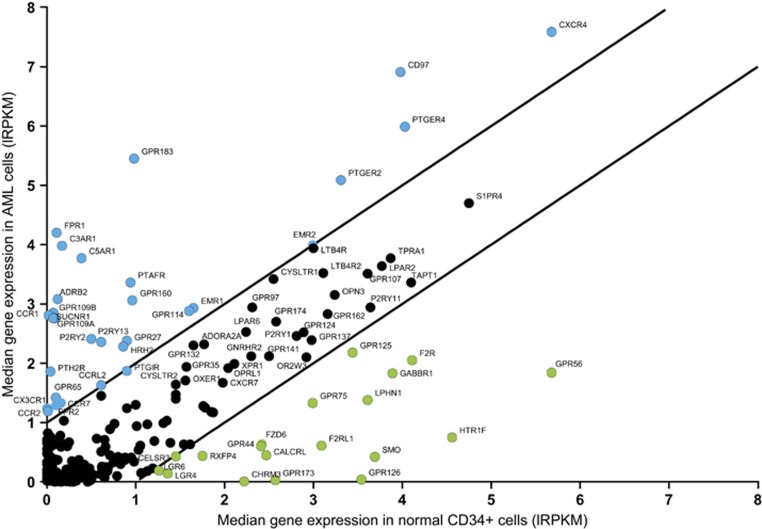
Relation between GPCR expression in AML and in normal cord blood-derived CD34^+^ cells. The median gene expression level of 772 GPCRs in AML cells (*y* axis) is represented against their expression in normal cord blood-derived CD34^+^ cells (*x* axis). The 30 upregulated GPCRs in AML (blue dots) have a difference in median expression level between AML and normal CD34^+^ cells greater than 1. The 19 downregulated GPCRs in AML (green dots) have a difference in median expression less than −1. GPCRs represented in black dots are not differentially expressed between AML and normal CD34^+^ cells. RNA-Seq data were transformed to lRPKM (log_2_(RPKM+1)).

When compared with normal cord blood-derived CD34^+^ cells, 30 GPCRs are upregulated in AML specimens (blue dots in Figure 2). The most highly expressed GPCRs in AML are *CXCR4*, *CD97*, *PTGER4*, *PTGER2*, *EMR2*, *GPR183*, *FPR1*, *C3AR1* and *C5AR1*. Except for *FPR1* and *C5AR1*, these GPCRs show little inter-specimen variability (Figure 1 and Supplementary Table 5). Likewise, 19 GPCRs are less expressed in AML cells (green dots in Figure 2). *GPR56*, *HTR1F*, *SMO* and *GPR126* are most discriminatory of normal CD34^+^ cells. Most GPCRs are equally expressed in AML and normal CD34^+^ cells (black dots in Figure 2).

**Figure 3:**
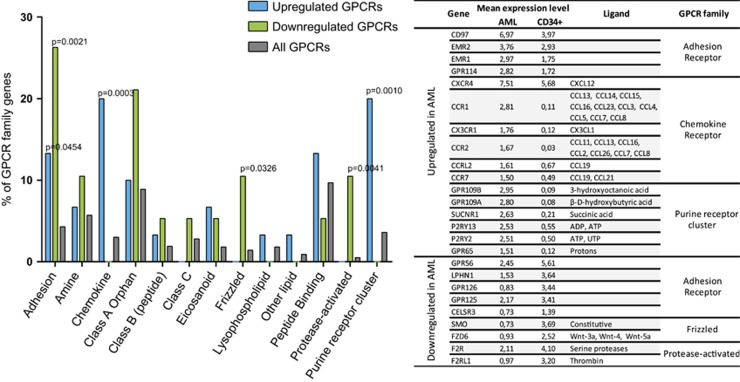
GPCR subfamily distribution of upregulated and downregulated GPCRs in AML. The left panel shows the proportion of genes upregulated or downregulated in AML and all GPCRs into different subfamilies of GPCRs (adhesion, amine, chemokine and so on). The *P*-values are indicated for significant families by Fisher's exact tests. The right panel shows individual GPCRs of enriched groups. Values indicated in second and third columns correspond to the receptor mean expression level in AML and CD34^+^ cells. All represented GPCRs have mean expression levels in AML significantly different from their mean in normal CD34^+^ cells, *P*-values<0.005. The Fisher's exact test was performed between the upregulated or downregulated group and remaining GPCRs, i.e., all GPCRs excluding differentially expressed genes.

Class enrichment analyses showed that both AML upregulated and downregulated GPCRs are enriched in adhesion GPCRs compared with their representation in the genome indicating that this subfamily of receptors is highly deregulated in AML compared with the overall GPCR family. Chemokine and purine receptors were overrepresented in AML upregulated genes, whereas protease-activated GPCRs and Frizzled family members were overrepresented among the AML downregulated transcripts indicating that these subclasses of receptors are disproportionally affected in the disease state (Figure 3).

### GPCRs are differentially expressed in distinct AML genetic subgroups

We next studied GPCR expression levels in relation to the most frequent AML genetic subgroups represented in this cohort, that are AML with t(8;21)(q22;q22), inv(16)(p13.1q22) or *MLL* translocations, and normal karyotype AML with *NPM1*, *DNMT3A* or *FLT3*-ITD mutations (Supplementary Table 2).

**Figure 4:**
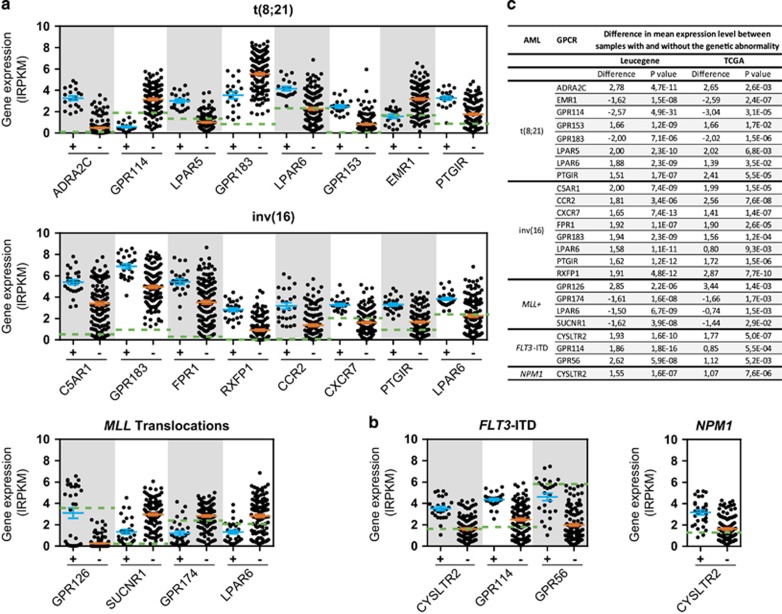
GPCR expression level analysis in AML of different genetic subgroups. Expression of deregulated GPCRs in AML samples with (**a**) t(8;21), inv(16) and *MLL* translocations and (**b**) normal karyotype with *FLT3*-ITD or *NPM1* mutations. Differentially expressed GPCRs are defined as having a difference in mean expression higher or equal to 1.5 lRPKM between samples with (+) and without (−) the genetic abnormality and a significant Student's *t* test (*P*<0.05). Only GPCRs that were validated in the TCGA dataset are shown. Data are expressed as individual sample expression value and means ±1 s.e.m. for all samples. Mean expression of GPCRs in normal CD34^+^ cells is illustrated with a green dashed line. (**c**) List of GPCRs with a significant difference in mean expression level in AML samples of representative genetic subgroups in Leucegene and TCGA cohorts. RNA-Seq data were transformed to lRPKM (log_2_(RPKM+1)).

A GPCR expression fingerprint was observed for AML samples with t(8;21), inv(16) and *MLL* translocations (Figure 4a and Supplementary Figure 6A). For example, eight GPCRs were specifically upregulated or downregulated in the AML subgroup with the t(8;21) translocation. These included the adrenergic receptor *ADRA2C*, the orphan receptor *GPR153* and the lipid receptors *LPAR5*, *LPAR6* and *PTGIR* (all upregulated) as well as the adhesion GPCRs, *EMR1* and *GPR114* and the oxysterol-binding receptor, *GPR183* (downregulated). Overexpression of eight other GPCRs occurs in the inv(16) AML subgroup. These are *C5AR1*, *CCR2*, *CXCR7/ACKR3*, *FPR1*, *GPR183*, *RXFP1*, *PTGIR* and *LPAR6* (Figure 4a). AML with *MLL* translocations were associated with an upregulation of *GPR126* and a downregulation of *GPR174*, *SUCNR1* and *LPAR6* (Figure 4a, bottom panel). In addition, *GPR126* expression differed between subtypes of *MLL* rearranged leukemias according to the translocation partners, being overexpressed at a high level in AML samples with the *MLL-MLLT4*, *MLL-ELL* and *MLL-SEPT9* fusions and not expressed in the majority of AML samples with the *MLL-MLLT3* and *MLL-MLLT1* fusions (Supplementary Figure 7). *FLT3*-ITD mutated samples revealed an upregulation of *CYSLTR2*, also observed in *NPM1*-mutated samples, and of the adhesion GPCRs, *GPR114* and *GPR56* (Figure 4b and Supplementary Figure 6B). These results were validated in an independent AML dataset of 160 samples available from The Cancer Genome Atlas (TCGA) project which comprised 7 samples with t(8;21), 12 with inv(16), 11 with *MLL* translocations and normal karyotype AML with *FLT3*-ITD (*n*=22), *NPM1* (*n*=43) or *DNMT3A* mutations (*n*=30) (Figure 4c). *DNMT3A*-mutated samples did not reveal any significant GPCR expression fingerprint when analyzed in the TCGA cohort.

**Figure 5:**
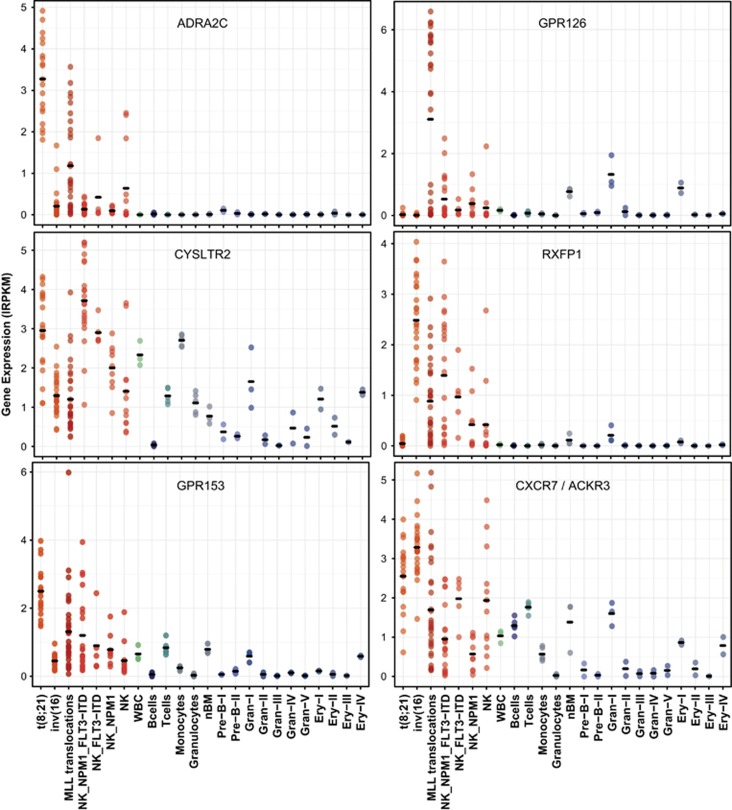
Expression levels of GPCRs overexpressed in specific AML genetic subgroups in AML, normal blood and bone marrow cells. Expression levels of GPCRs previously identified as overexpressed in specific genetic subgroups of AML are compared with their expression in normal blood and bone marrow cell populations. Represented GPCRs, i.e., *ADRA2C* and *GPR153* in AML with t(8;21), *GPR126* in AML with *MLL* translocations, *CYSLTR2* in normal karyotype AML with *NPM1* or *FLT3*-ITD mutations, and *RXFP1* and *CXCR7/ACKR3* in AML with inv(16), show a significant difference in mean expression between the specific AML genetic subgroup and each normal cell population identified (Student's *t* test (*P*<0.05)). AML samples are represented by red dots and normal cell samples by green, blue or gray dots. Data are expressed as individual sample expression value and means for all samples. Normal blood and bone marrow cell populations were prepared as described in the Materials and methods section. RNA-Seq data were transformed to lRPKM (log_2_(RPKM+1)). NK, normal karyotype; WBC, white blood cells; nBM, normal bone marrow.

Ideal therapeutic targets should be expressed on leukemic cells but not on normal bone marrow and blood hematopoietic cells. Accordingly, we analyzed the genes upregulated in genetic subgroups by comparing their expression in AML cells with expression levels in normal mature blood cells and bone marrow erythroid, myeloid and B-cell precursors. Interestingly, *ADRA2C*, *GPR126*, *CYSLTR2*, *RXFP1*, *GPR153* and *CXCR7/ACKR3* maintained their significant overexpression in specific AML genetic subgroups when compared with normal cell populations (Figure 5 and Supplementary Table 9).

## Discussion

Our results revealed that 30 GPCRs are overexpressed in AML samples compared with normal CD34^+^ cells. These receptors are enriched in the chemokine (*CCR1*, *CXCR4*, *CCR2*, *CX3CR1*, *CCR7* and *CCRL2*), adhesion (*CD97*, *EMR1*, *EMR2* and *GPR114*) and purine (including *P2RY2* and *P2RY13*) receptor subfamilies. This list includes GPCRs previously described as important for AML cell biology, such as *CXCR4*,^24^ as well as GPCRs that have a role in hematopoietic stem cell engraftment, such as *C3AR1* and *PTGER2*.^25, 26^ In addition, 19 receptors are downregulated in AML cells including adhesion GPCRs like *LPHN1*, *GPR125*, *GPR56*, *CELSR3* and *GPR126*, protease-activated receptors (*F2R* and *F2RL1*) and the Frizzled family members *SMO* and *FZD6*.

Among these deregulated GPCRs, prostaglandin receptors are of particular interest. Indeed, both *PTGER4* and *PTGER2* are among the most highly expressed GPCRs differentiating AML from normal CD34^+^ cells. Preliminary results have shown that PGE2 can increase cyclic AMP production by human leukemic cells through EP2 (encoded by *PTGER2*) but not EP4 receptors. Further functional studies are needed to confirm these observations.^27^ Interestingly, highly selective EP2 antagonists (TG4-155 and TG6-10-1) have recently been developed and might therefore be available for pre-clinical studies.^28^ It is worth noting that different studies have highlighted the importance of PGE2, acting through four GPCRs, *PTGER1–4*, in the progression of many cancers including colorectal, gastric, lung or breast cancers.^29^

Chemokine receptors found to be overexpressed in AML specimens such as *CXCR4*, *CCR7*, *CCR1*, *CCR2*, *CX3CR1* and *CCRL2* are also of potential interest. Except for CXCR4,^24^ their role in AML has never been invoked. In other hematological cancers, CX3CR1 and its ligand, CX3CL1, have been proposed to be involved in the interaction between chronic lymphocytic leukemia cells and their microenvironment,^30^ and CCR1 has a crucial role in the pathogenesis of myeloma-associated bone disease. Interestingly, CCX721, a selective CCR1 inhibitor, improves osteolytic bone lesions in a preclinical mouse model of this disease.^31^ This compound is analogous to CCX354-C, an oral CCR1 antagonist that has been evaluated in clinical trial for human rheumatoid arthritis.^32^

The purine receptor family members overexpressed in AML include *GPR109A*, *GPR109B*, *SUCNR1*, *P2RY2*, *P2RY13* and *GPR65*. To our knowledge, none of these receptors have been involved previously in hematologic malignancies. However, *GPR109A* is silenced in colon cancer and primary breast tumor tissues where it acts as a tumor suppressor.^33^ In contrast to these findings, the receptor is overexpressed in squamous cell cancers compared with normal keratinocytes.^34^ *P2RY2* mediates prostate cancer cell migration and metastasis,^35^ and its activation leads to increased proliferation of melanoma^36^ and lung tumor cells.^37^ This observation is context-dependent because *P2RY2* activation induces apoptosis in human colorectal carcinoma cell lines.^38^ The pH-sensing receptor *GPR65* has also been reported to be overexpressed in a significant proportion of kidney, ovarian, colon and breast tumors.^39^ Recent findings have also highlighted that a member of this GPCR family, P2Y14, is critical for stress hematopoiesis.^40^

Adhesion GPCRs are a family of receptors characterized by a long extracellular domain and an autoproteolytic site leading to an extracellular N-terminal fragment and a transmembrane C-terminal fragment that remain noncovalently associated after cleavage. These receptors have been shown to have a crucial role in development, cell migration and are increasingly being invoked as deregulated in numerous cancers.^41^ They represent another group of GPCRs for which we observed a strong expression in AML. Notably, *CD97* is expressed at high levels in 100% of AML studied to date at a much higher level than found in normal CD34^+^ cells. *CD97* is upregulated in a variety of other malignancies, including glioblastoma,^42^ digestive and thyroid cancers.^43^ *GPR56* and *GPR114* are specifically overexpressed in *FLT3*-ITD-mutated samples and *GPR126* is most specific to AML with *MLL* translocations. Notably, *GPR56* has recently been identified as a leukemia stem cell marker, and its expression correlates with poor prognosis in AML.^44, 45^

Two protease-activated GPCRs *F2R* or *PAR1* and *F2RL1* or *PAR2* are downregulated in AML. *PAR1* has a well-established role in thrombosis, hemostasis and inflammatory diseases. It is also involved in promoting growth and in the angiogenesis and metastasis processes of several malignancies.^4^ Unlike AML samples, most solid tumors show an upregulation of *PAR1* expression. Our findings are in accordance with the results of a recent study that demonstrated that *PAR1* expression is downregulated in primary AML cells.^46^ Furthermore, *Par1* homozygous null mutant mice develop leukemia with a shorter latency than control animals in secondary transplantation experiments.^46^ *PAR2* is involved in migration and/or proliferation of many cancer cells, including breast,^47^ pancreatic^48^ or colon^49^ cancer and seems to have a role in tumor angiogenesis through vascular endothelial growth factor production in cancer cells.^50^

Two frizzled class receptors *SMO* and *FZD6* are also downregulated in all AML samples except AML with *EVI1* rearrangements, which have a *FZD6* median expression close to CD34^+^ cells. SMO and Patched are two receptors mediating Hedgehog signaling. This pathway is aberrantly activated in many solid tumors including basal cell carcinoma.^51^ This feature has been exploited for the development of Vismodegib, an antagonist of SMO.^13^ However, in contrast to several other cancers, *SMO* expression in AML cells is five times lower than in CD34^+^ cells in our study. Vismodegib is currently being investigated in a phase II clinical trial (www.clinicaltrials.gov, NCT02073838) in AML patients in combination with ribavirin, because it could potentially overcome the resistance of leukemic cells to ribavirin.^52^

It is tempting to speculate that the GPCR members that are differently expressed in frequent AML genetic subgroups and more specifically expressed in AML cells compared with normal hematopoietic cells could be exploited for the development of novel therapeutic approaches. In this context, *ADRA2C*, which is upregulated in AML with t(8;21), could be an interesting target to explore, because many agonists and antagonists of this receptor are available, including several FDA-approved drugs such as the antihypertensive drug clonidine and the antidepressant Mirtazapine. *CYSLTR2*, a receptor for the inflammatory mediators cysteinyl leukotrienes, is also overexpressed in *FLT3*-ITD- and *NPM1-*mutated samples. In aggressive breast tumors, this gene has been described as an independent prognostic factor in combination with *CYSLTR1*.^53^ Selective agonist (NMLTC4)^54^ and antagonists, such as HAMI3379^(ref. 55)^ or BayCysLT2,^56^ have been developed for CYSLTR2. Other interesting targets are the GPCR members that are overexpressed in AML with inv(16) or AML M4 (Supplementary Table 8). For example, selective antagonists of the chemokine receptor CCR2, such as CCX140-B, studied in diabetic mice and tested in clinical trials for patients with diabetic nephropathy (www.clinicaltrials.gov, NCT01447147 and NCT01440257) might be explored for anti-leukemic activity.^57^

In conclusion, we provide the first comprehensive transcriptome analysis of GPCRs in AML, which reveals that these surface receptors are potential novel therapeutic targets in AML. Using available agonists/antagonists to leukemia-enriched GPCRs, specific drugs can now be tested in preclinical models and, when active, in clinical trials for AML therapeutics.
