# Supplementary material for: Transcriptome analysis of G protein-coupled receptors in distinct genetic subgroups of acute myeloid leukemia: identification of potential disease-specific targets
Source: Blood Cancer J. 2016 Jun 3;6(6):e431–. doi: 10.1038/bcj.2016.36 (PMC5141352; doi:10.1038/bcj.2016.36)
Supplement: Supplementary Information [file bcj201636x1.pdf]

Transcriptome analysis of G Protein-Coupled Receptors in distinct genetic subgroups of acute myeloid leukemia: identification of potential disease-specific targets

Arhamatoulaye Maiga *et al*.

Supplementary information

Supplementary methods 2

Plasmids 2

Antibody validation, cell culture and transfection 2

Flow cytometry analysis of selected highly expressed GPCRs 2

Flow cytometry for sorting of normal bone marrow and peripheral blood cell populations 2

Quantitative RT-PCR experiments 3

List of primers and probes used for quantitative RT-PCR 3

Legends to supplementary tables 3

Suppl. Table 1. Characteristics of the 148 Leucegene AML samples. 3

Suppl. Table 2. Genetic subgroups of AML samples analyzed. 4

Suppl. Table 3. Sorting strategy for normal bone marrow cell populations. 4

Suppl. Table 4. GPCR classification in subfamilies.. 4

Suppl. Table 5. Expression of 772 GPCRs in 148 Leucegene AML samples. 4

Suppl. Table 6. Expression of 772 GPCRs in 12 samples of cord blood-derived $CD34^+$ cells. 4

Suppl. Table 7. Expression of 10 selected GPCRs and 4 control genes in 10 AML samples using quantitative RT-PCR. 5

Suppl. Table 8. GPCR expression level analysis in M4 and M5 AML morphologic subtypes. 5

Suppl. Table 9. Expression levels of GPCRs overexpressed in specific AML genetic subgroups in AML, normal blood and bone marrow cells 5

Legends to supplementary figures 5

Suppl. Fig. 1. Threshold definition for (A) highly expressed GPCRs and (B) GPCRs with low variability in their expression. 5

Suppl. Fig. 2. Identification of overexpressed GPCRs in cord blood-derived $CD34^+$ cells 6

Suppl. Fig. 3. RNA-Seq data validation of GPCR expression in AML samples by quantitative RT-PCR 6

Suppl. Fig. 4. Validation of antibodies for flow cytometry analysis of selected GPCRs 7

Suppl. Fig. 5. Flow cytometry analysis of selected highly expressed GPCRs 7

Suppl. Fig. 6. GPCRs with deregulated expression in at least one frequent genetic subgroup. 7

Suppl. Fig. 7. *GPR126* expression in AML samples with different *MLL* translocation partners. 8
References……………………………………………………………………………………..8

Supplementary methods

Plasmids

CD97 cDNA inserted in pCMV SPORT6 vector was obtained from Open Biosystems (MHS4768-99609488) and CXCR4, FPR1 or C5AR1 in pcDNA3.1 was obtained from the Missouri S&T cDNA Resource Center (www.cdna.org). LTB4R expressed in pJ3M has been previously described in (Gaudreau, Le Gouill et al. 1998).

Antibody validation, cell culture and transfection

The HEK293 cell line which has a low expression level of LTB4R, FPR1 and C5AR1 was used for antibody validation. Antibodies were validated by comparing their staining pattern between untransfected cells and cells transfected with a vector containing the corresponding cDNA. HEK293 cells were cultured in 6-well plates in Dulbecco's Modified Eagle's Medium (DMEM) supplemented with 10% (v/v) FBS at 37°C with 5% CO2. At 80% of confluence, cells were transfected with 1µg of plasmid DNA encoding a GPCR by using Lipofectamine 2000 (Invitrogen). Cell were harvested at 48h after transfection, and suspended in 100 µL PBS buffer before staining with the antibodies.

Flow cytometry analysis of selected highly expressed GPCRs

Commercial antibodies used for GPCR detection in AML or HEK293 cells were CXCR4-PerCP (BioLegend, #306515), CD97-PE (BioLegend, #336307), LTB4R-PE (LS Bio, #LS-C16203), FPR1-APC (R&D system, #FAB3744A) and C5AR1-FITC (LS Bio, #LS-C5674). We used the LSR II equipment (BD Biosciences) for flow cytometry data acquisition, and the DIVA or FlowJo software for analysis.

Flow cytometry for sorting of normal bone marrow and peripheral blood cell populations

The following anti-human antibodies were used for sorting of normal bone marrow and peripheral blood populations: CD34 FITC (BD 555821), CD71 SPC (BD 334108), Gpa PerCP eFluor710 (eBioscience 46-9987-42), CD10 BV421 (BD 562902), CD15 FITC (BD 555401), CD33 PE (BD 555450), CD34 APC (BD 555824), CD11b PE-Cy5 (BD 555389), CD16 Pacific Blue (BD

558122), CD13 APC-Cy7 (BioLegend 301710), CD3 FITC (BD 555332), CD14 APC-Cy7 (BD 560919), CD19 APC-Cy7 (BioLegend 302218), CD19 PE-Cy7 (BD 557835). Cells were stained for 30 minutes at 4°C. Fc-blocking reagent (BD 564219) was used to minimize non-specific binding of antibodies.

Quantitative RT-PCR experiments

Total RNA was extracted from AML cells using TRIzol reagent (Invitrogen). Reverse transcription for cDNA generation using MMLV reverse transcriptase was performed according to the manufacturer's instructions (Invitrogen). Quantitative PCR experiments were performed in 384-well plates in 10µL final volume using the TaqMan Fast Advanced Master Mix of Applied Biosystems. A mix of the forward and reverse oligos at 250 nM final each, 100nM of the UPL probe (Universal probe library from Roche) and the cDNA was added to 1X of the TaqMan Fast Mix. All reactions were done in duplicates. The amplification was processed with the following protocol: 3min at 95°C, followed by 40 cycles at 95°C 5sec and 60°C 30sec on a thermal cycler 7900HT (Applied Biosystems). Data were analysed using the SDS 2.4 and Data Assist softwares. Delta CTs were obtained from Ct gene – Ct reference using 4 control genes (*GAPDH*, *ACTB*, *EIF4H*, *HNRNPL*).

List of primers and probes used for quantitative RT-PCR

GENE	Forward primer	Reverse primer	UPL probe
C3AR1	gccttcagctactgtctcagttt	gagaattactgggggctcatt	76
CD97	gaagctgaattgggctgtg	gttctggatggagaggatgc	78
FPR1	aagaccacagctggtgaaca	atacagcaggtgtccctcca	68
GPR126	cttccactgtgctatgaaggag	tggtagctgtcttactccaatctg	70
GPR183	gacccgaacgagtcactgat	cagagggcggagtaaaattg	77
LPAR6	cacaaacatttgttaattgctcagt	cagagagtgattgggtacattgtc	74
LTB4R	gactccaacttcctgccaat	aggaaggcatcccctagaac	55
OPN3	gggccattacctacatctgg	gtccaggatgtacctgttcca	51
PTGER4	ctccctggtggtgctcat	ggctgatataactggttgacga	58
TAPT1	ctctgtagcacggaggatgg	ccttgcactttaattgagcttg	55

Legends to supplementary tables

Suppl. Table 1. Characteristics of the 148 Leucegene AML samples.

NPM1, *DNMT3A* or *FLT3*-ITD mutated AML samples are indicated by 1 and samples without the mutation by 0.

Suppl. Table 2. Genetic subgroups of AML samples analyzed.

Suppl. Table 3. Sorting strategy for normal bone marrow cell populations.
Subpopulations were sorted based on surface marker combinations described by (Novershtern, Subramanian et al. 2011).

Suppl. Table 4. GPCR classification in subfamilies

The GPCR classification was based on the IUPHAR database. To complete and subdivide the class A group, the GRAFS phylogenetic classification of GPCRs was also used. Taste receptors, which are not included in the IUPHAR database, have been added as well as vomeronasal receptors, opsins and 3 orphan GPCRs (GPR137B, TAPT1, XPR1). GPCRs were classified in 18 sub-families.

Suppl. Table 5. Expression of 772 GPCRs in 148 Leucegene AML samples.

RNA-Seq was used to determine expression levels of 772 GPCRs in 148 AML samples of the Leucegene cohort. Data are represented for individual samples and classified by their median expression value in AML. NK, Normal Karyotype; Int. Abn., Intermediate abnormal karyotype; MLL+, AML with *MLL* translocations; EVI1+, AML with *EVI1* rearrangements; Complex, AML with 3 or more unrelated clonal chromosomal abnormalities. FAB, French-American-British classification. The GPCR subclass and ligand, the average and median level of expression as well as the coefficient of variation are indicated for each GPCR analyzed. A t-test was performed to compare the mean expression level of GPCRs in AML and normal $CD34^+$ cells. The number of AML samples with an expression level of ≥1 lRPKM is indicated for each GPCR. RNA-Seq data were transformed to lRPKM (log_2(RPKM+1)). RPKM: Reads Per Kilobase per Million mapped reads.

Suppl. Table 6. Expression of 772 GPCRs in 12 samples of cord blood-derived $CD34^+$ cells.

RNA-Seq was used to determine expression levels of 772 GPCRs in 12 samples of normal cord blood-derived $CD34^+$ $CD45RA^-$ cells. Data are represented for individual samples and classified by their median expression value in $CD34^+$ cells. The GPCR subclass and ligand, the average and median level of expression as well as the coefficient of variation are indicated for each GPCR analyzed. RNA-Seq data were transformed to lRPKM (log_2(RPKM+1)). RPKM: Reads Per Kilobase per Million mapped reads.

Suppl. Table 7. Expression of 10 selected GPCRs and 4 control genes in 10 AML samples using quantitative RT-PCR.

Expression levels of 10 selected GPCRs and 4 control genes using RNA-Seq are transformed to lRPKM (log2(RPKM+1)). The delta CT values (CT of tested GPCR – mean CT of 4 control genes) represent a mean of 2 independent quantitative RT-PCR experiments. RPKM: Reads Per Kilobase per Million mapped reads.

Suppl. Table 8. GPCR expression level analysis in M4 and M5 AML morphologic subtypes. RNA-Seq was used to determine expression levels of 772 GPCRs in AML samples with a monocytic component (FAB M4 and M5 subtypes) and compared to their expression in all other AML samples of the Leucegene cohort. RNA-Seq data were transformed to lRPKM (log_2(RPKM+1)). Overexpressed GPCRs in AML M4 or M5 are defined as having a difference in mean expression higher or equal to 1.5 lRPKM between AML M4 or M5 samples and other AML samples and a significant student's t test ($p < 0.05$). Results obtained for GPCR members identified in the table have been validated in the TCGA dataset. GPCRs identified in bold are also overexpressed in AML with inv(16). FAB, French-American-British classification; RPKM: Reads Per Kilobase per Million mapped reads.

Suppl. Table 9. Expression levels of GPCRs overexpressed in specific AML genetic subgroups in AML, normal blood and bone marrow cells.

Expression levels of *ADRA2C*, *GPR153*, *CXCR7/ACKR3*, *RXFP1*, *GPR126* and *CYSLTR2* in AML specimens of specific genetic subgroups and in normal cell samples are shown. The student's t-test was calculated between specific AML genetic subgroup and each normal cell sample identified. RNA-Seq data were transformed to lRPKM (log2(RPKM+1)). RPKM: Reads Per Kilobase per Million mapped reads.

Legends to supplementary figures

Suppl. Fig. 1. Threshold definition for (A) highly expressed GPCRs and (B) GPCRs with low variability in their expression.

The mean expression level or the coefficient of variation (CV) (X axis) is represented for each of the 772 GPCRs analyzed in this study. Each dot represents a GPCR after classification by (**A**) decreasing median level of expression or (**B**) increasing CV (Y axis). The grey area delimited

GPCRs considered (**A**) to be highly expressed or (**B**) to have a low variability in their expression among AML samples. RNA-Seq data were transformed to lRPKM (log_2(RPKM+1)). Highly expressed GPCRs were selected using a threshold of 3.5 lRPKM (or 10.35 RPKM). The threshold for GPCR with low variability in their expression was established at a CV of 50%. RPKM: Reads Per Kilobase per Million mapped reads.

Suppl. Fig. 2. Identification of overexpressed GPCRs in cord blood-derived $CD34^+$ cells.

RNA-Seq was used to determine expression levels of 772 GPCRs in 148 AML samples of the Leucegene cohort and 12 samples of normal cord blood-derived $CD34^+$ $CD45RA^-$ cells. The 50 GPCRs with the highest median expression levels in cord blood-derived $CD34^+$ cells are presented in the heatmap. The GPCRs which have a higher expression level as defined in Supplementary Fig. 1A are in bold. NK, Normal Karyotype; Int.abn., Intermediate abnormal karyotype; MLL+, AML with *MLL* translocations; EVI1+, AML with *EVI1* rearrangements; Complex, AML with 3 or more unrelated clonal chromosomal abnormalities. Boxes in green, blue or grey represents one sample with *NUP98-NSD1* fusion, 17p deletion or an insufficient number of metaphases respectively. RNA-Seq data were transformed to lRPKM (log_2(RPKM+1)). RPKM: Reads Per Kilobase per Million mapped reads.

Suppl. Fig. 3. RNA-Seq data validation of GPCR expression in AML samples by quantitative RT-PCR.

Expression levels of 10 selected GPCRs obtained by the real-time quantitative PCR method were compared to RNA-Seq data. RNA-Seq values were transformed in lRPKM (log_2(RPKM+1)). The 10 GPCRs tested include highly expressed members such as *PTGER4*, *GPR183* and *CD97* (mean expression level above 5 lRPKM), weakly expressed GPCRs such as *GPR126* (0.8 lRPKM), and GPCRs with an intermediate level of expression such as *LPAR6* (2.5 lRPKM). For normalization of qPCR data, 4 endogenous control genes were used, including *GAPDH* and *ACTB* and 2 recently described control genes, *EIF4H* and *HNRNPL* (Macrae, Sargeant et al. 2013) (Supplementary Table 7). The correlation between results obtained from qPCR experiments (Delta CT, Y axis) and RNA-Seq analysis (lRPKM, X axis) was 0.94 ($p<0.0001$). Each dot represents a mean of 2 independent experiments. The Pearson's R correlation coefficient varies from -0.79 to -0.98, supporting a robust correlation between the two datasets. Delta Ct represents the difference

between the Ct of the tested gene and the mean Ct of 4 endogenous control genes. RPKM: Reads Per Kilobase per Million mapped reads.

Suppl. Fig. 4. Validation of antibodies for flow cytometry analysis of selected GPCRs.

HEK293 cells were transfected with CXCR4, CD97, LTB4R, FPR1 or C5AR1 cDNA. Transfected or untransfected cells were stained with respective antibodies for flow cytometry analysis. Positive population of transfected cells (blue) was compared to the untransfected cells (red) using their respective unstained cells as control (green and orange). For the five receptors tested, a clear difference of staining between these cells confirms that these antibodies target the expected proteins.

Suppl. Fig. 5. Flow cytometry analysis of selected highly expressed GPCRs.

The protein expression of 5 selected GPCRs was assessed in 20 AML samples of different morphologic subtypes by flow cytometry analysis. (A) Representative histogram of one AML sample (03H041) following staining with antibodies for CD97, LTB4R, CXCR4, C5AR1, or FPR1. Red histograms represent unstained AML cells used as controls (B) Dot plot representing the percentage of protein-expressing cells observed in each AML specimen stained. Variable protein expression for CXCR4 (10 to 84% positive cells), LTB4R (0 to 95% positive cells), FPR1 (0 to 69% positive cells), and C5AR1 (1 to 95% positive cells) is demonstrated. CD97 shows strong expression in all cells of each of the 20 studied AML samples (92.5 to 99.6 % positive cells).

Suppl. Fig. 6. GPCRs with deregulated expression in at least one frequent genetic subgroup.

Expression of deregulated GPCRs in AML samples with (**A**) t(8;21), inv(16), normal karyotype (NK) and *MLL* translocations and (**B**) normal karyotype with *DNMT3A*, *FLT3*-ITD or *NPM1* mutations. Differentially expressed GPCRs were identified in the Leucegene cohort and are defined as having a difference in mean expression higher or equal to 1.5 lRPKM between samples with (+) and without (-) the genetic abnormality and a significant student's t test ($p<0.05$). GPCRs are identified in blue if they are validated in the TCGA dataset and in yellow if not confirmed in the TCGA cohort. Grey boxes represent differences of expression that are not significant. RNA-

Seq values were transformed to lRPKM (log_2(RPKM+1)). RPKM: Reads Per Kilobase per Million mapped reads.

Suppl. Fig. 7. *GPR126* expression in AML samples with different *MLL* translocation partners.

GPR126 expression levels are represented in the Y axis and the different translocation partners of the Leucegene AML samples with *MLL* translocations are represented in the X axis. Data are expressed as individual sample expression value and means +/- 1 SEM for all samples. RNA-Seq data were transformed to lRPKM (log_2(RPKM+1)). RPKM: Reads Per Kilobase per Million mapped reads; SEM: standard error of the mean.

References

Gaudreau, R., C. Le Gouill, S. Metaoui, S. Lemire, J. Stankova and M. Rola-Pleszczynski (1998). "Signalling through the leukotriene B4 receptor involves both alphai and alpha16, but not alphaq or alpha11 G-protein subunits." Biochem J **335 (Pt 1)**: 15-18.

Macrae, T., T. Sargeant, S. Lemieux, J. Hebert, E. Deneault and G. Sauvageau (2013). "RNA-Seq reveals spliceosome and proteasome genes as most consistent transcripts in human cancer cells." PLoS One **8**(9): e72884.

Novershtern, N., A. Subramanian, L. N. Lawton, R. H. Mak, W. N. Haining, M. E. McConkey, N. Habib, N. Yosef, C. Y. Chang, T. Shay, G. M. Frampton, A. C. Drake, I. Leskov, B. Nilsson, F. Preffer, D. Dombkowski, J. W. Evans, T. Liefeld, J. S. Smutko, J. Chen, N. Friedman, R. A. Young, T. R. Golub, A. Regev and B. L. Ebert (2011). "Densely interconnected transcriptional circuits control cell states in human hematopoiesis." Cell **144**(2): 296-309.
