# Supplementary material for: Transcriptome analysis of G protein-coupled receptors in distinct genetic subgroups of acute myeloid leukemia: identification of potential disease-specific targets
Source: Blood Cancer J. 2016 Jun 3;6(6):e431–. doi: 10.1038/bcj.2016.36 (PMC5141352; doi:10.1038/bcj.2016.36)

Suppl. Figure 1

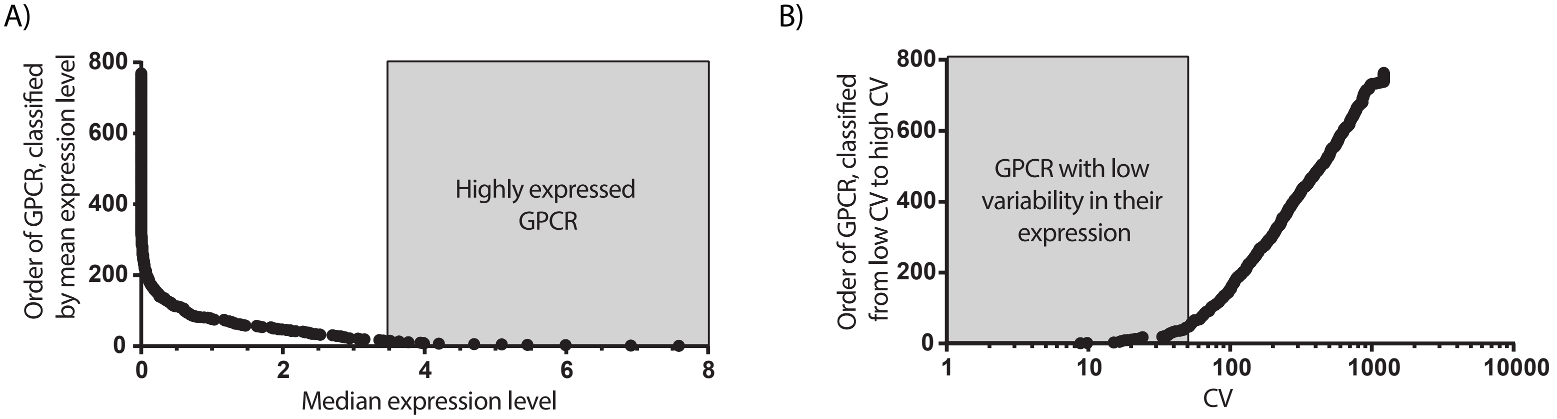

Suppl. Figure 2

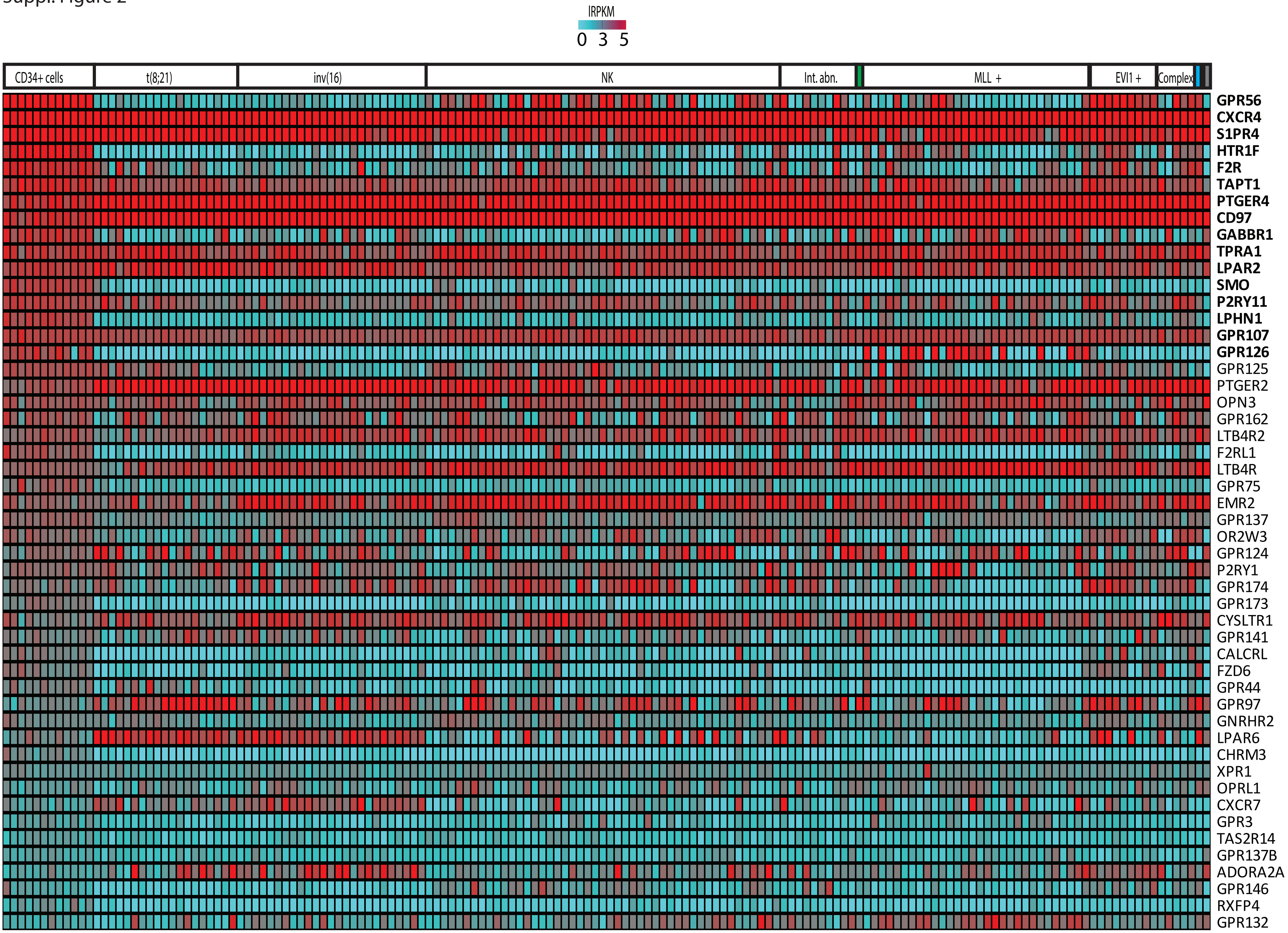

Suppl. Figure 3

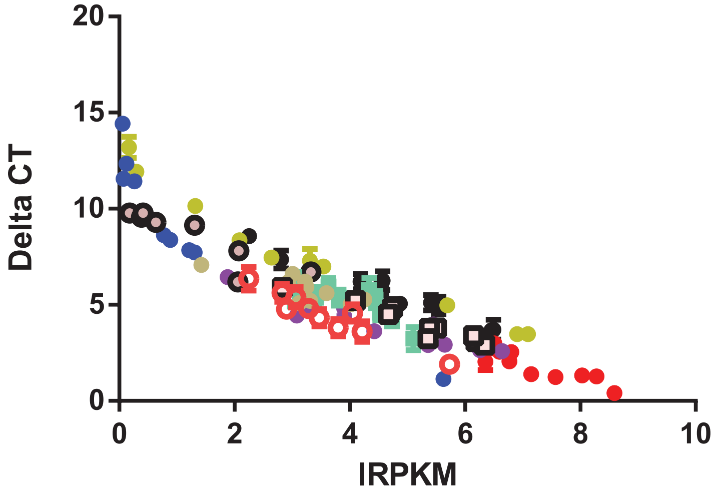

Gene	Pearson R	P value
C3AR1	-0.98	< 0.0001
CD97	-0.87	0.0010
FPR1	-0.97	< 0.0001
GPR126	-0.91	0.0002
GPR183	-0.93	< 0.0001
LPAR6	-0.93	< 0.0001
LTB4R	-0.80	0.0054
OPN3	-0.79	0.0060
PTGER4	-0.96	< 0.0001
TAPT1	-0.96	< 0.0001

Suppl. Figure 4

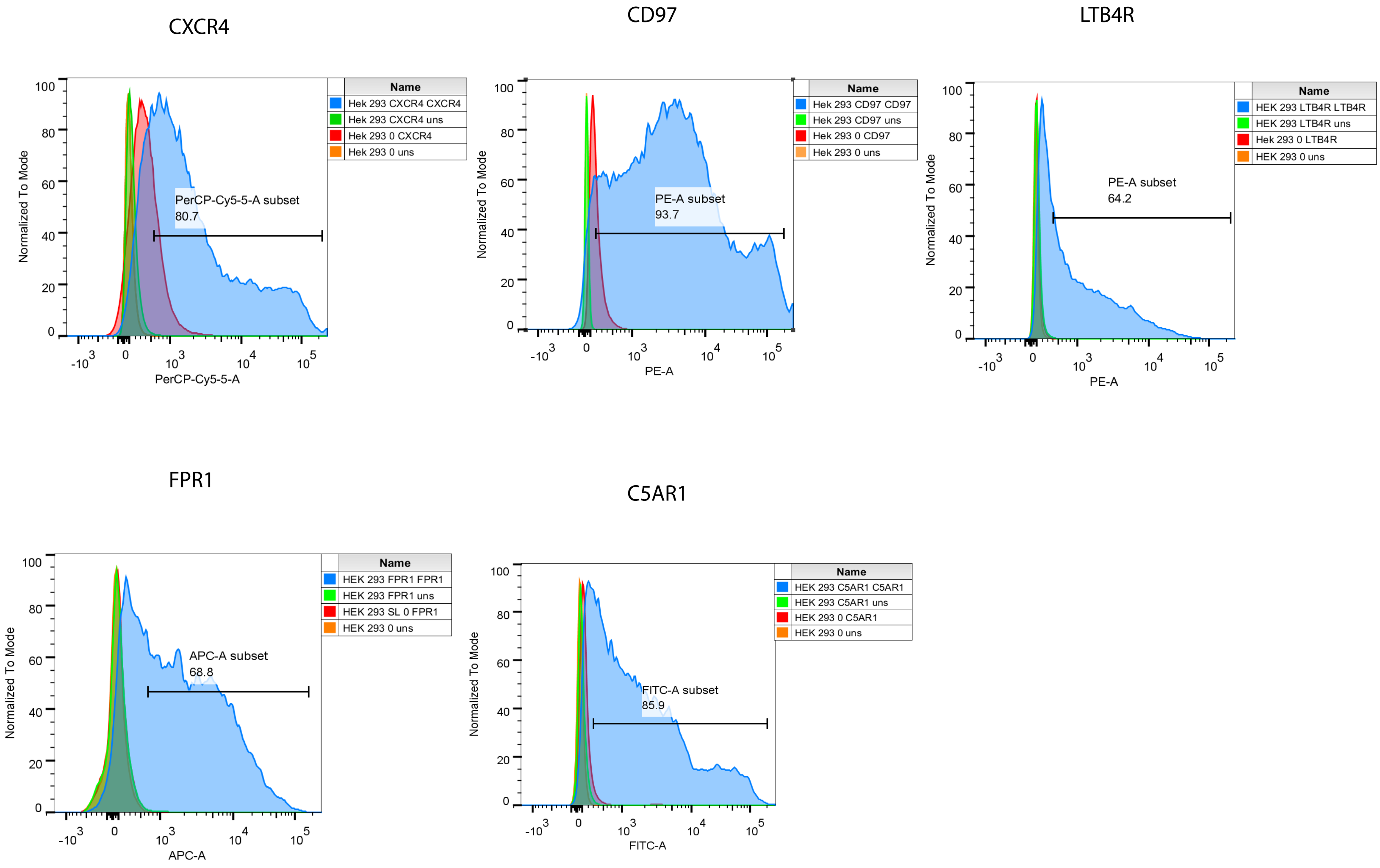

Suppl. Figure 5

A)

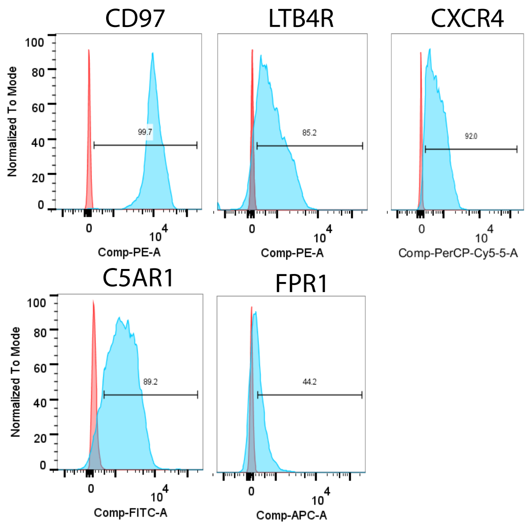

B)

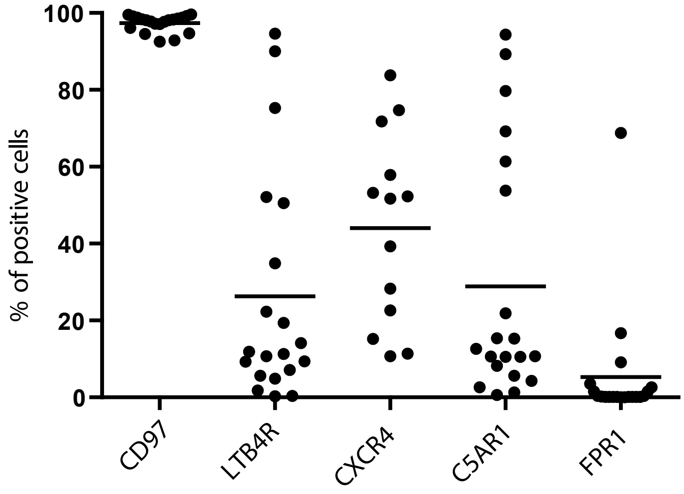

Suppl. Figure 6

A)

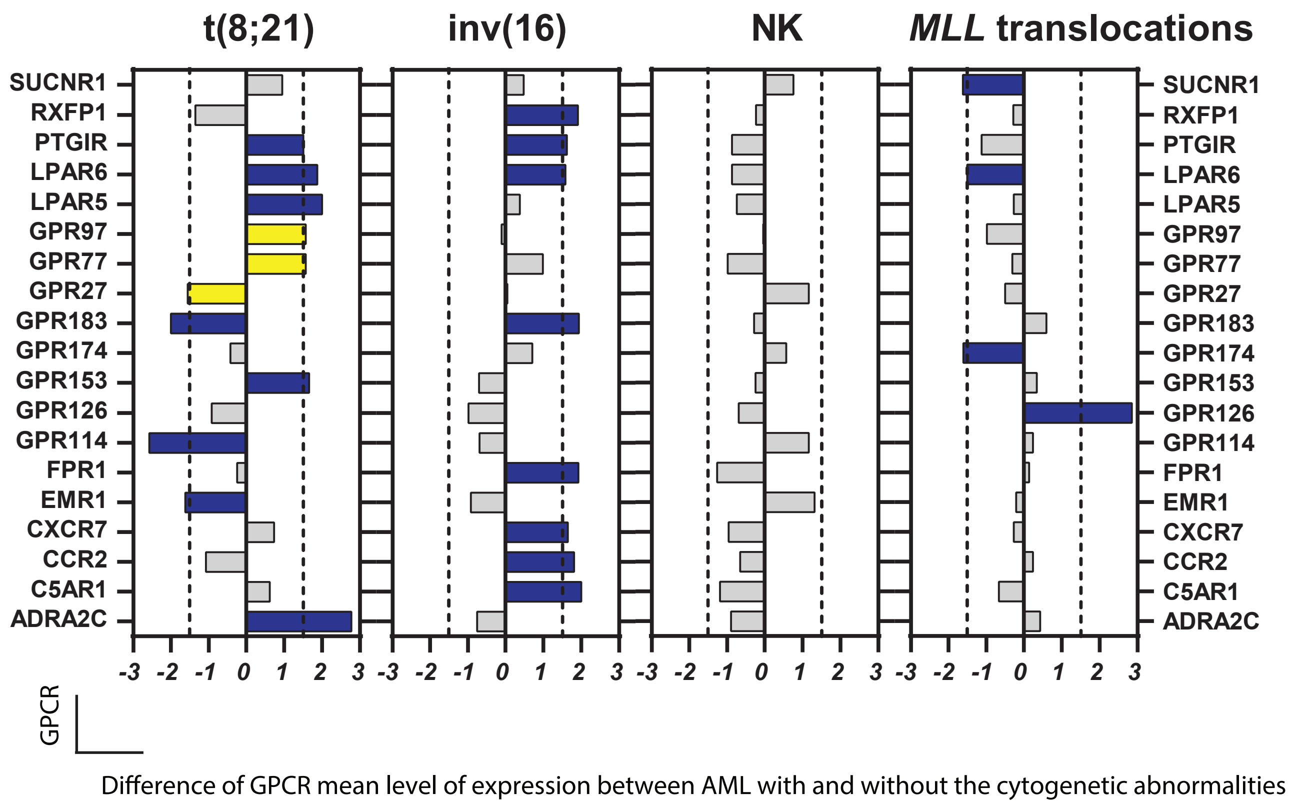

B)

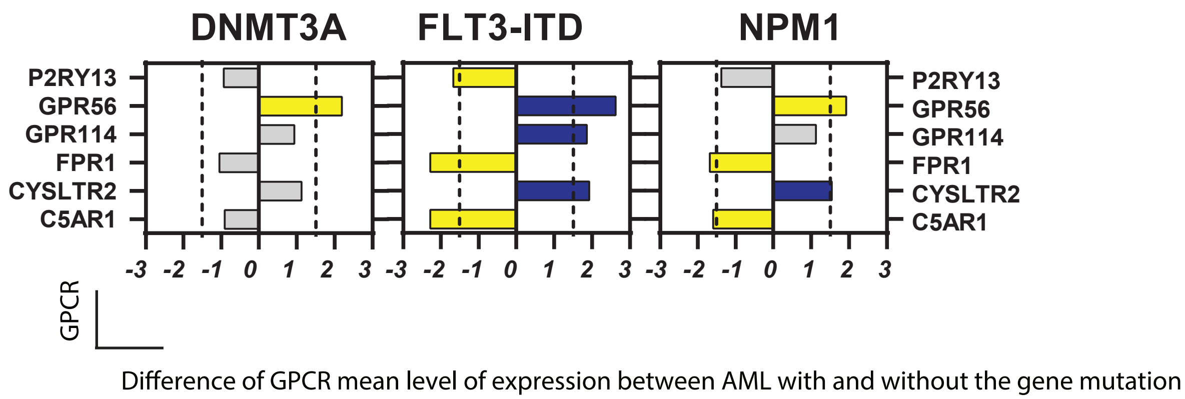

Suppl. Figure 7

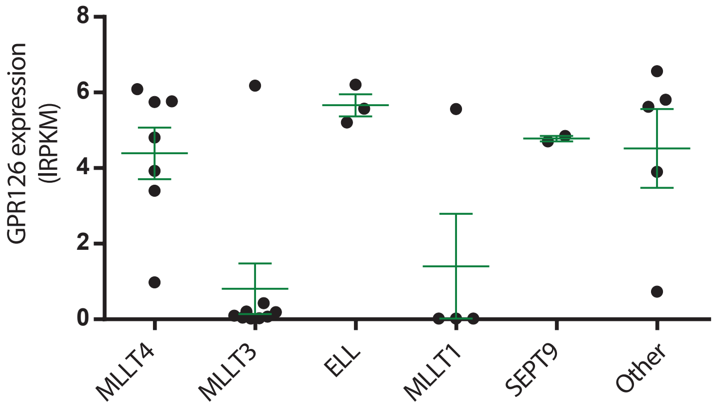

Supplement: Supplementary Figures [file bcj201636x2.pdf]
