# Supplementary material for: Transcriptome analysis of G protein-coupled receptors in distinct genetic subgroups of acute myeloid leukemia: identification of potential disease-specific targets
Source: Blood Cancer J. 2016 Jun 3;6(6):e431–. doi: 10.1038/bcj.2016.36 (PMC5141352; doi:10.1038/bcj.2016.36)
Supplement: Supplementary Tables 2–4 [file bcj201636x4.pdf]

Supplementary Table 2 Genetic subgroups of the AML samples analyzed

**Cytogenetic subgroups of AML**	**Number of samples**
t(8;21)(q22;q22)	19
inv(16)(p13.1q22)	25
normal karyotype	47
MLL translocations	30
intermediate abnormal karyotype	10
EVI1 rearrangements	9
complex karyotype and abnormalities of chromosome 17p	6
NUP98-NSD1 fusion	1
insufficient number of metaphases	1
**Gene mutation in AML with normal karyotype**	
NPM1	30
DNMT3A	26
FLT3 -ITD	26

Supplementary Table 3. Sorting strategy for normal bone marrow cell populations

cocktail	cell type	FITC	PE	APC	PE-Cy5	PerCP-efluor710	PacBlue	BV421	APC-Cy7
**cocktail 1**	Pre-B-I	CD34+						CD10+	CD19+
	Pre-B-II	CD34-						CD10+	CD19+
	Ery-I	CD34+		CD71+		Gpa-			
	Ery-II	CD34-		CD71+		Gpa-			
	Ery-III	CD34-		CD71+		Gpa+			
	Ery-IV	CD34-		CD71-		Gpa+			
**cocktail 2**	Gran-I	CD15-	CD33+	CD34+	CD11b-		CD16-		CD13+
	Gran-II	CD15+	CD33+	CD34-	CD11b-		CD16-		
	Gran-III	CD15+	CD33+	CD34-	CD11b+		CD16-		CD13-
	Gran-IV	CD15+	CD33+	CD34-	CD11b+		CD16med		
	Gran-V	CD15+	CD33+	CD34-	CD11b+		CD16+++		CD13+

Suppl. Table 4

GPCRs class	subclass	Classification	Database	number of GPCRs
Class A	Non olfactory	Adenosine	IUPHAR	4
		Amine biogene	IUPHAR and GRAFS	44
		Chemokine	IUPHAR and GRAFS	23
		Class A orphan	IUPHAR + 5 opsin	69
		Eicosanoid	IUPHAR and GRAFS	14
		Glycoprotein	IUPHAR and GRAFS	3
		Lysophospholipid	IUPHAR	14
		Other lipid	IUPHAR	7
		Peptide binding	IUPHAR	75
		Protease	IUPHAR	4
		Purine cluster	GRAFS	28
	Olfactory	Olfactory	GRAFS (370 olfactory + 4 Vomeronasal)	374
Adhesion		Adhesion	IUPHAR	33
Class B		Class B	IUPHAR	15
Class C		Class C	IUPHAR	22
Frizzled		Frizzled	IUPHAR	11
Taste		Taste	GRAFS	24
Other 7 TM		Other 7 TM	IUPHAR + GPR137B, TAPT1 and XPR1	8
